# The Canadian Joint Replacement Registry—what have we learned?

**DOI:** 10.3109/17453671003685467

**Published:** 2010-03-31

**Authors:** Eric R Bohm, Michael J Dunbar, Robert Bourne

**Affiliations:** ^1^Canadian Joint Replacement Registry and Section of Orthopedic Surgery, University of Manitoba, WinnipegLondon; ^2^Canadian Joint Replacement Registry and Section of Orthopedic Surgery, Dalhousie University, HalifaxLondon; ^3^Canadian Joint Replacement Registry and Section of Orthopedic Surgery, University of Western OntarioLondon

## Abstract

The Canadian Joint Replacement Registry (CJRR) was launched in 2000 through the collaborative efforts of the Canadian Orthopedic Association and the Canadian Institutes for Health Information. Participation is voluntary, and data collected by participating surgeons in the operating room is linked to hospital stay information from administrative databases to compile yearly reports. In the fiscal year 2006–2007, there were 62,196 hospitalizations for hip and knee replacements in Canada, excluding Quebec. This represents a 10-year increase of 101% and a 1-year increase of 6%. Compared to men, Canadian women have higher age-adjusted rates per 105 for both TKA (148 vs. 110) and THA (86 vs. 76). There also exist substantial inter-provincial variations in both age-adjusted rates of arthroplasty and implant utilization that cannot be explained entirely on the basis of differing patient demographics. The reasons for these variations are unclear, but probably represent such factors as differences in provincial health expenditure, efforts to reduce waiting lists, and surgeon preference. The main challenge currently facing the CJRR is to increase procedure capture to > 90%. This is being pursued through a combination of efforts including simplification of the consent process, streamlining of the data collection form, and the production of customized reports with information that has direct clinical relevance for surgeons and administrators. As the CJRR continues to mature, we are optimistic that it will provide clinically important information on the wide range of factors that affect arthroplasty outcome.

## Introduction

Through the collaborative efforts of the Canadian Orthopedic Association (COA) and the Canadian Institutes for Health Information (CIHI), the Canadian Joint Replacement Registry (CJRR) was launched in June 2000. A strategic decision was made by the framers of the CJRR to partner with the CIHI, an independent national repository for health information, to allow linkage of CJRR data to other longitudinal databases held by the CIHI. It was also appreciated that a collaborative arrangement would afford a stronger voice for the CJRR to interact with provincial and federal funders of Canadian healthcare.

## Structure

**Figure 1.**
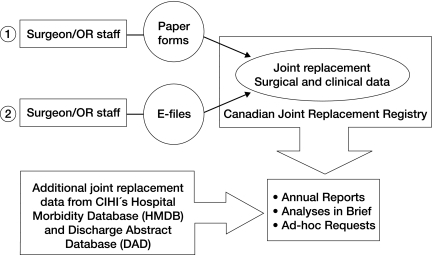
Canadian Joint Replacement Registry Data Flow Diagram (adapted with permission from the CJRR 2009 report).

The basic registry data is collected by the surgeon (or appointee) in the operating room on a 2-sided standardized form that records patient demographics, implant information, and information on surgical technique (see supplementary data). The surgeon then has the option to send the completed form to CIHI in either an electronic or paper format. Patients are also asked to sign a consent form permitting the collection, linking, and analysis of their health information. Once collected, these core registry data are then combined with additional CIHI data from both the Hospital Morbidity Database (“HMDB”, which contains national data on hospital inpatient events) and the Discharge Abstract Database (“DAD”, which contains national data on hospital discharges) to produce both the yearly reports and “analysis in brief” reports (Figure 1).

## Governance

The leadership of the CJRR is informed by an advisory committee consisting of a chairperson (an orthopedic surgeon), orthopedic representatives from each province, representatives from the Arthritis Society of Canada, patients, nursing staff, and members of the CIHI assigned to the CJRR project (18 individuals in total). The committee meets in person at least once a year at the COA annual meeting; this is augmented with national conference calls at least once a year.

A Research and Development sub-committee, consisting of orthopedic surgeons, is charged with defining research questions of interest, assessing requests for data analysis, and assisting in defining the content of the Annual Report and Analyses in Brief documents. The Research and Development committee also meets in person once a year at the annual COA meeting and by teleconference, as necessary.

As custodian of the data, the CIHI is responsible for safeguarding the privacy and confidentiality of both providers' and patients' information. The CIHI's mandate includes the production and dissemination of information to be used for Canadian health system management, and it is empowered to do so without explicit consent from patients. In addition to releasing the regular CJRR reports, the CIHI responds to ad hoc requests by third parties, all in accordance with the CIHI's privacy policy.

Currently, patient consent is sought for CJRR participation; however, in certain jurisdictions, provincial or territorial privacy legislation may allow for the data to be sent to CIHI without explicit patient consent, provided the purpose is for health system management.

## Funding

Funding for the CJRR is provided to the CIHI by Health Canada and provincial and territorial governments.

## Data capture

Participation is not mandatory, but 70% of orthopedic surgeons in Canada who perform arthroplasty state that they participate in the registry (Canadian Institute for Health Information 2008). For the fiscal year 2006–2007, CJRR forms were submitted on only 41% of hip and knee arthroplasty cases—an obvious shortfall in data collection. A recent survey of COA members designed to identify barriers to participation indicated that personally completing and submitting both the registry form and patient consent form to CIHI were important issues. Recent CIHI data show that consent is missing on 31% of the forms submitted to the CJRR. Completed data forms without consent are not currently entered into the database (Canadian Institute for Health Information 2009). These factors, combined with recent changes in provincial healthcare data privacy legislation that would no longer require patient consent for this type of health database, may result in the consent form being abolished (personal communication with Brent Diverty, director of the CIHI).

## Observations from the annual CJRR reports

The CJRR Annual Reports are available online in PDF format at www.cihi.ca/cjrr. In the fiscal year 2006–2007, there were 62,196 hospitalizations for hip and knee replacements in Canada, excluding Quebec. This represents a 10-year increase of 101% and a 1-year increase of 6%. The 10-year increase, and in particular the 1-year increase of 17% that occurred in 2005–6, are a result of national efforts announced in 2005 that focused on reducing waiting times for hip and knee replacements (Canadian Intergovernmental Conference Secretariat).

Compared to men, Canadian females have higher age-adjusted rates per 10^5^ for both TKA (148 vs. 110) and THA (86 vs. 76). Accordingly, women account for 57% of patients receiving THA and 61% of patients receiving TKA. Using CJRR data, a strong relationship between obesity and subsequent risk of undergoing both THA and TKA has been demonstrated (Bourne et al. 2007). Interestingly, the largest increases in both TKA and THA rates occurred in the 45–65-year age groups, where the greatest increases in Canadian obesity rates have also occurred (Tjepkema 2009).

**Figure 2.**
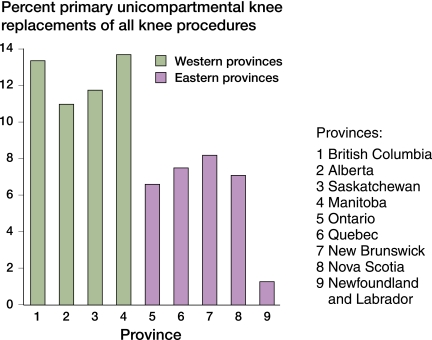
Primary unicompartmental knee replacements – by Province 2004–2005.

Provision of healthcare in Canada is the individual responsibility of each of the 13 provinces and territories. In return for federal funding, each province or territory must provide universal healthcare that meets the provisions of the federal Health Care Act. The Act outlines requirements for public administration, portability, comprehensiveness, accessibility, and universality. This structure has essentially resulted in 13 different (but similar) healthcare plans across Canada, each with different priorities and funding levels. The differences between each province and territory are highlighted by findings in the CJRR reports: differences by province exist in patient demographics and implant selection that cannot be explained on the basis of patient differences alone. For example, in 2006, the use of unicompartmental knee arthroplasty (UKA) varied from a low of 1% in Newfoundland to a high of 14% in Manitoba (Canadian Institute for Health Information 2007) (Figure 2). The demographics of the 2 provinces are too similar (Statistics Canada 2008) for this to explain the variation in use of UKA. Perhaps it is a result of direct-to-consumer advertising (Bozic et al. 2007) spilling into Canada from other jurisdictions?

In a similar fashion, age-adjusted rates of THA for men range from a low of 45 per 10^5^ in Newfoundland to a high of 94 per 10^5^ in Saskatchewan; age-adjusted TKA rates for women range from a low of 84 per 10^5^ in Newfoundland to a high of 183 per 10^5^ in Manitoba. These differences represent a variation of greater than 100%, and cannot be explained on the basis of patient factors alone: the rate of obesity and thus the theoretical risk of requiring arthroplasty (Bourne et al. 2007) is actually higher in Newfoundland than in either Manitoba or Saskatchewan (Tjepkema 2009).

The CJRR also highlights some of the differences in the delivery of arthroplasty care around the world. In Sweden, the incidence of TKA in females for 2006–2007 was 136 per 10^5^, and in Canada it was 182 per 10^5^. While the differences in arthroplasty rates between the 2 countries are certainly multifactorial, it is conceivable that the higher rates of TKA in Canada may be related to its higher rates of obesity (OECD 2005) (Tjepkema 2009). Other interesting differences include THA stem fixation methods. The National Joint Registry of England and Wales (2008) reports a lower early revision rate for cemented stems than for cementless stems (0.7% vs 1.8%, p < 0.01); yet in Canada, the use of cementless THA stems continues to grow: in 2006–2007 it stood at 71%. The reasons for this difference have not been clearly delineated, but possible explanations include a lack of survivalship data in the Canadian registry on cemented and cementless THA stems, efforts to reduce surgical waiting times by increasing operating room throughput (using cementless stems removes the “cement curing time” from the overall operative time), and advertising directed at surgeons and patients in the US that makes its way into Canada.

## Challenges and future directions

Data capture with CJRR forms remains a challenge: only 41% of all TKAs and THAs had forms submitted to the CIHI in 2006–7. The CJRR is taking a multi-pronged approach to address this deficiency by focusing on several areas that have been identified as being problematic: consent, data collection form, and relevance. Privacy legislation is being reviewed on a province-by-province basis to see whether the consent form can be abolished altogether, as many provinces allow collection of data for purposes of healthcare system improvement without explicit patient consent. Furthermore, the data collection form will most likely undergo revisions to align it with the 14 data elements suggested by the International Society of Arthroplasty Registries (Robertsson 2007); this should make form completion by OR staff possible. CIHI is working with its advisory committee to develop focused, clinically relevant comparative reports that surgeons, provinces, and hospitals can use to compare themselves to their peer groups. It is hoped that this type of concrete feedback will encourage participation and further facilitate improvement of care. The CJRR is also pursuing the use of Discharge Abstract Database and Hospital Morbidity Database information to develop survivorship curves for procedures that were captured in the CJRR database via the submission form but may not have had a form submitted for the revision procedure. If successful, this will allow the development of survivorship curves for the many different data elements that the CJRR form collects—ranging from specific implant types, the addition of antibiotic to cement, to surgical approach, and many others (supplementary data, CJRR data collection forms).

We are optimistic that these continued efforts will improve data capture and enable even higher quality information to be provided to Canadian surgeons, hospitals, and healthcare administrators on the many factors that affect arthroplasty outcomes.

## Supplementary Appendix

Supplementary Appendix is available at our website (www.actaorthop.org), identification number 3790/10.
