# Supplementary material for: The Canadian Joint Replacement Registry—what have we learned?
Source: Acta Orthop. 2010 Mar 31;81(1):119–21. doi: 10.3109/17453671003685467 (PMC2856215; doi:10.3109/17453671003685467)
Supplement: Supplementary file 1 [file ORT-1745-3674-81-119-s3790.pdf]

Canadian Institute for Health Information
Institut canadien d'information sur la santé

For CIHI use only

Unique Identifier

# CANADIAN JOINT REPLACEMENT REGISTRY

## *Knee Replacement Data Collection Form*

Addressograph

Surgeon First Name

Surgeon Last Name

***Has Patient Consent Been Obtained?*** ☐ **Yes** ☐ **No** **If no, please complete only surgeon first/last name and hospital name, and forward to CIHI.**

Patient First Name | Middle Initial | Patient Last Name

Provincial Health Card Number

**Province Code**

Birth Date Y Y Y Y M M D D

Home Postal Code

Gender ☐ Male ☐ Female

Height (cm) •

Weight (kg) •

Hospital Chart Number

Hospital Province

Hospital Name ______________________________

Surgery Date Y Y Y Y M M D D

Admission Date (if different from surgery date) Y Y Y Y M M D D

## *Wait Time Information*

Referral Date **(New Patients Only)** Y Y Y Y M M D D

Date of First Consult **(New Patients Only)** Y Y Y Y M M D D

Date of Decision for Surgery **(All Patients)** Y Y Y Y M M D D

***Please Complete This Form by Checking (√) the Appropriate Box (es)***

| | |
|---|---|
| **Side (Location)** | ***If bilateral, complete ONE form PER SIDE***<br>Unilateral ☐ Right ☐ Left Bilateral ☐ Right ☐ Left |
| **Type of Replacement** | ***Check ONE only:***<br>☐ Primary ☐ R1 ☐ R2 ☐ R3 ☐ >R3 ☐ Excision (not a revision) |
| **Diagnosis Grouping** (for primary replacement only) | ***Check MOST RESPONSIBLE diagnosis to involved knee:***<br>☐ Degenerative OA ☐ Inflammatory Arthritis ☐ Post Traumatic OA ☐ Osteonecrosis<br>☐ Infection ☐ Tumour ☐ Acute fracture ☐ Other ______________ |
| **Reason(s) for Revision**<br>☐ N/A or ➔ ➔ | ***Check ALL that apply to involved knee:***<br>☐ Aseptic Loosening ☐ Infection—Single Stage ☐ Implant Fracture ☐ Poly Wear ☐ Instability<br>☐ Pain of Unknown Origin ☐ Infection—Two Stage ☐ Bone Fracture ☐ Osteolysis ☐ Unispacer<br>☐ Unresurfaced Patella ☐ Patella Maltracking ☐ Patella Failure ☐ Other ______________ |
| **Previous Operations**<br>☐ None or ➔ ➔ | ***Check ALL that apply to involved knee:***<br>☐ Total Knee Arthroplasty ☐ Unicompartmental Arthroplasty ☐ Tibial Osteotomy ☐ Femoral Osteotomy<br>☐ Open Menisectomy ☐ Arthroscopic Menisectomy ☐ Patellectomy ☐ Ligament Repair<br>☐ Fracture Fixation ☐ Arthroscopic Debridement ☐ Other ______________ |
| **Joint Deformity**<br>☐ None or ➔ ➔ | ***Check ALL that apply to involved knee:***<br>☐ Varus ☐ Valgus ☐ Flexion contracture ☐ If Any ≥15° |
| **Surgical Approach** | ***Check APPROACH:***<br>☐ Medial Parapatellar ☐ Lateral Parapatellar ☐ Intravastus ☐ Subvastus ☐ Other ______________<br>***Minimally Invasive (MIS)?*** ☐ Yes ☐ No |
| **Special Steps**<br>☐ None or ➔ ➔ | ***Check ALL that apply to involved knee:***<br>☐ Lateral Retinacular Release ☐ Rectus Snip ☐ Quadriceps Turndown<br>☐ Tubercle Osteotomy ☐ Other ______________ |
| **Antibiotic Use** | ***Will antibiotics be administered prophylactically?*** ☐ Yes ☐ No<br>***If yes, duration?*** ☐ ≤24 hours ☐ >24 hours |
| **DVT Prevention** | ***Will DVT prophylaxis be given in hospital?*** ☐ Yes ☐ No<br>***If yes, check ALL that apply:*** ☐ Warfarin ☐ LMW Heparin ☐ ASA ☐ SC Heparin<br>☐ Pneumatic Stockings ☐ Foot Pump ☐ Other ______________ |
| **O.R. Environment** | ***Check ALL that apply:*** ☐ Laminar Air Flow ☐ No Laminar Air Flow ☐ Body Exhaust ☐ Ultraviolet |

**CANADIAN JOINT REPLACEMENT REGISTRY** **KNEE REPLACEMENT DATA COLLECTION FORM**

**Femoral Component**

**Replaced?**
☐ Yes →
☐ No ↓

**Unicompartmental Prosthesis?**
☐ Yes ☐ No

**If yes,**
☐ Medial ☐ Lateral ☐ Patellofemoral

**Cemented?** ☐ Yes ☐ No

**If Cemented:**
Porosity Reduction: ☐ Yes ☐ No

**If Cementless, *Check ALL that apply*:**
☐ Porous In-growth ☐ H. A. Coated
☐ Press-fit

**Manufacturer** See Legend Below
Femoral Component ☐

*Affix sticker(s) or catalogue and lot number beside the specific component.*
*Do not include stickers for screws.*

Sticker for Femoral Component

**Tibial Component**

**Replaced?**
☐ Yes →
☐ No → ↓

**Unicompartmental Prosthesis?**
☐ Yes ☐ No

**If yes,**
☐ Medial ☐ Lateral ☐ Patellofemoral

**All Polyethylene?** ☐ Yes ☐ No
**Mobile Bearing?** ☐ Yes ☐ No
**Cemented?** ☐ Yes ☐ No

**If Cemented:**
Porosity Reduction? ☐ Yes ☐ No

**If Cementless, *Check ALL that apply*:**
☐ Porous In-growth Screws? ☐ Yes ☐ No
☐ H.A. Coated Screws? ☐ Yes ☐ No
☐ Trabecular Metal Screws? ☐ Yes ☐ No

**PCL Retaining** Or **PCL Sacrificing**
☐ Standard ☐ Post & Cam ☐ Constrained
☐ Medial Pivot ☐ Deep Dished ☐ Hinged
☐ Medial Pivot

Manufacturer
Poly Insert ☐
Tibial Base Plate ☐

Sticker(s) for Poly (Insert)

Sticker(s) for Tibial Base Plate

**Patellar Component**

**Resurfaced?**
☐ Yes →
☐ No ↓

**Cemented?** ☐ Yes ☐ No

Type: ☐ Inset ☐ Onlay

Manufacturer
Patellar Component ☐

Sticker for Patellar Component

**Modular Stem**
☐ Yes →
☐ No ↓

| **Stems** | **Femoral** | **Tibial** |
|---|---|---|
| Cemented | ☐ | ☐ |
| Cementless | ☐ | ☐ |

**Metal Augments**
☐ Yes →
☐ No ↓

| **Augments** | **Femoral** | **Tibial** |
|---|---|---|
| Metal | ☐ | ☐ |
| Trabecular metal | ☐ | ☐ |

Manufacturer
Femoral Stem ☐
Tibial Stem ☐

Sticker(s) for Modular Femoral Stems

Sticker(s) for Modular Tibial Systems

**Cement Details**

**Cement Used?**
☐ Yes →
☐ No ↓

***For each item, Check ONE only:***

**Cement Name:**
☐ Simplex ☐ Zimmer ☐ CMW ☐ Versabond
☐ Palacos ☐ Osteobond ☐ Cerafix ☐ Other ____________

**Antibiotics Added by Manufacturer?** ☐ Yes ☐ No
**Antibiotics Added by Surgeon?** ☐ Yes ☐ No
***If Yes*, Antibiotic Name:**
☐ Tobramycin ☐ Vancomycin ☐ Gentamicin ☐ Erythromycin ☐ Other
**Mixing Method:** ☐ Vacuum-mixed ☐ Centrifuge ☐ Manually Mixed

Sticker for Cement Type

**Bone Graft Used—Femur**
☐ Yes →
☐ No ↓

***Check ALL that apply:***

| | Autograft | | | Allograft | Bone Graft Substitute |
|---|---|---|---|---|---|
| | Iliac crest | Local bone | Other | | |
| Structural | ☐ | ☐ | ☐ | ☐ | ☐ |
| Strut/Onlay | ☐ | ☐ | ☐ | ☐ | ☐ |
| Morselized | ☐ | ☐ | ☐ | ☐ | ☐ |

**Bone Graft Used—Tibial**
☐ Yes →
☐ No ↓

***Check ALL that apply:***

| | Autograft | | | Allograft | Bone Graft Substitute |
|---|---|---|---|---|---|
| | Iliac crest | Local bone | Other | | |
| Structural | ☐ | ☐ | ☐ | ☐ | ☐ |
| Strut/Onlay | ☐ | ☐ | ☐ | ☐ | ☐ |
| Morselized | ☐ | ☐ | ☐ | ☐ | ☐ |

**Legend of Manufacturer Codes:**
01 Biomet
02 Ceraver
03 J&J/DePuy
04 Sulzer/Centerpulse
05 Zimmer
06 Wright Medical
07 Smith & Nephew Richards
08 Stryker/Osteonic/Howmedica
99 Other
