# Supplementary material for: The Canadian Joint Replacement Registry—what have we learned?
Source: Acta Orthop. 2010 Mar 31;81(1):119–21. doi: 10.3109/17453671003685467 (PMC2856215; doi:10.3109/17453671003685467)
Supplement: Supplementary file 2 [file ORT-1745-3674-81-119-s3790_2.pdf]

Canadian Institute for Health Information
Institut canadien d'information sur la santé

For CIHI use only

Unique Identifier

CANADIAN JOINT REPLACEMENT REGISTRY
Hip Replacement Data Collection Form

Addressograph

Surgeon First Name

Surgeon Last Name

Has Patient Consent Been Obtained? ☐ Yes ☐ No **If no, please complete only surgeon first/last name and hospital name, and forward to CIHI.**

Patient First Name | Middle Initial | Patient Last Name

Provincial Health Card Number

Province Code

Birth Date Y Y Y Y M M D D

Home Postal Code

Gender ☐ Male ☐ Female

Patient's Height (cm) •

Weight (kg) •

Hospital Chart Number

Hospital Province

Hospital Name ______________________

Surgery Date Y Y Y Y M M D D

Admission Date (If different from surgery date) Y Y Y Y M M D D

Wait Time Information

Referral Date **(New Patients Only)** Y Y Y Y M M D D

Date of First Consult **(New Patients Only)** Y Y Y Y M M D D

Date of Decision for Surgery **(All Patients)** Y Y Y Y M M D D

Please Complete This Form by Checking (√) the Appropriate Box(es)

**Side (Location)**	***If bilateral, complete ONE form PER SIDE***<br>Unilateral ☐ Right ☐ Left Bilateral ☐ Right ☐ Left
**Type of Replacement**	***Check ONE only:***<br>☐ Primary ☐ R1 ☐ R2 ☐ R3 ☐ >R3 ☐ Excision (not a revision)
**Diagnosis Grouping** (for primary replacement only)	***Check MOST RESPONSIBLE diagnosis to involved hip:***<br>☐ Degenerative OA ☐ Inflammatory Arthritis ☐ Post Traumatic OA ☐ Osteonecrosis<br>☐ Childhood Hip Problem ☐ Old Hip Fracture ☐ Acute Hip Fracture ☐ Tumour<br>☐ Infection ☐ Other ______________
**Reason(s) for Revision**<br>☐ N/A **or** ➔ ↓	***Check ALL that apply to involved hip:***<br>☐ Aseptic Loosening ☐ Infection—Single Stage ☐ Infection—Two Stage ☐ Poly Wear<br>☐ Osteolysis ☐ Instability ☐ Implant Fracture ☐ Bone Fracture<br>☐ Pain of Unknown Origin ☐ Leg length discrepancy ☐ Other ______________
**Previous Operations**<br>☐ None **or** ➔ ↓	***Check ALL that apply to involved hip:***<br>☐ Fracture Fixation ☐ Surface Replacement ☐ Pelvic Osteotomy ☐ Femoral Osteotomy<br>☐ Arthrodesis ☐ Resection Arthroplasty ☐ Hemiarthroplasty ☐ Total Hip Arthroplasty<br>☐ Other ______________
**Surgical Approach**	***Check APPROACH:***<br>☐ Smith/Peterson ☐ Anterolateral ☐ Posterolateral ☐ Direct Lateral ☐ 2-Incision ☐ Other ______________<br>***Minimally Invasive (MIS)?*** ☐ Yes ☐ No
**Special Techniques**<br>☐ None **or** ➔ ↓	☐ Trans-trochanteric (includes trochanteric slide) ☐ Extended Trochanteric ☐ Other ______________
**Antibiotic Use**	***Will antibiotics be administered prophylactically?*** ☐ Yes ☐ No<br>***If yes, duration?*** ☐ ≤24 hours ☐ >24 hours
**DVT Prevention**	***Will DVT prophylaxis be given in hospital?*** ☐ Yes ☐ No<br>***If yes, check ALL that apply:*** ☐ Warfarin ☐ LMW Heparin ☐ ASA ☐ SC Heparin<br>☐ Pneumatic Stockings ☐ Foot Pump ☐ Other ______________
**O.R. Environment**	***Check ALL that apply:*** ☐ Laminar Air Flow ☐ No Laminar Air Flow ☐ Body Exhaust ☐ Ultraviolet

CANADIAN JOINT REPLACEMENT REGISTRY **HIP REPLACEMENT DATA COLLECTION FORM**

Component	Details	Manufacturer	Sticker
**Femoral Component**<br>**Replaced/ Resurfaced?**<br>☐ Yes ➔<br>☐ No 🡣	**Surface Replacement?** ☐ Yes ☐ No<br>**Cemented?** ☐ Yes ☐ No<br>**If Cemented:**<br>Porosity Reduction: ☐ Yes ☐ No<br>**If Cementless, *Check ALL that apply*:**<br>☐ Porous In-growth ☐ H. A. Coated<br>☐ Grit Blasted<br>**Modular Stem?** ☐ Yes ☐ No	**Manufacturer** See Legend Below<br>Femoral Component ☐	*Affix sticker(s) or catalogue and lot number beside the specific component. Do not include stickers for screws.*<br>Sticker for Femoral Component
**Femoral Head**<br>**Replaced?**<br>☐ Yes ➔<br>☐ No 🡣	**Modular Head?** ☐ Yes ☐ No<br>**Outside Diameter:** ________ mm<br>**Material:**<br>☐ Cobalt Chrome ☐ Ceramic Zirconia<br>☐ Ceramic Alumina ☐ Oxinium<br>☐ Other ☐ Stainless steel<br>**Hemiarthroplasty?** ☐ Yes ☐ No<br>***If Yes,*** ☐ **Monopolar** ☐ **Bipolar**	Manufacturer<br>Femoral Head ☐	Sticker for Femoral Head
**Acetabular Component**<br>**Replaced?**<br>☐ Yes ➔<br>☐ No ➔ 🡣	**Cemented?** ☐ Yes ☐ No<br>**If Cemented:**<br>Same cement used for femoral component?<br>☐ Yes ☐ No<br>**If Cementless, *Check ALL that apply*:**<br>☐ Porous In-growth Screws? ☐ Yes ☐ No<br>☐ H.A. Coated Screws? ☐ Yes ☐ No<br>☐ Trabecular metal Screws? ☐ Yes ☐ No<br>Outside Diameter: ________ mm<br>**Tripolar Revision?** ☐ Yes ☐ No<br>**Constrained Cup?** ☐ Yes ☐ No	Manufacturer<br>Acetabular Component ☐	Sticker for Acetabular Component
**Acetabular Insert/Liner Replaced?**<br>☐ Yes ➔<br>☐ No ➔ 🡣	**Material: *(Check ONE only)***<br>☐ Polyethylene standard ☐ Ceramic Alumina<br>☐ Polyethylene cross-linked ☐ Metal<br>☐ Other<br>**Modular?** ☐ Yes ☐ No	Manufacturer<br>Acetabular Liner ☐	Sticker for Acetabular Liner
**Ring/Cage Used?**	☐ Yes ☐ No	Manufacturer<br>Ring/Cage ☐	Sticker for Acetabular Ring/Cage

Trabecular Metal Augment for Acetabulum used? ☐ Yes ☐ No

Cement	Details	Sticker
**Cement Details (Femoral Component)**<br>**Cement Used?**<br>☐ Yes ➔<br>☐ No 🡣	***For each item, Check ONE only:***<br>**Cement Name:**<br>☐ Simplex ☐ Zimmer ☐ CMW ☐ Versabond<br>☐ Palacos ☐ Osteobond ☐ Cerafix ☐ Other ____________<br>**Antibiotics added by manufacturer?** ☐ Yes ☐ No<br>**Antibiotics added by surgeon?** ☐ Yes ☐ No<br>***If Yes,*** **Antibiotic Name:**<br>☐ Tobramycin ☐ Vancomycin ☐ Gentamicin ☐ Erythromycin ☐ Other<br>**Mixing Method:** ☐ Vacuum-mixed ☐ Centrifuge ☐ Manually Mixed	Sticker for Cement Type

Bone Graft Used—Femur ☐ Yes ➔ ☐ No 🡣

Check ALL that apply:

	Autograft: Iliac crest	Autograft: Femoral head	Autograft: Other	Allograft	Bone Graft Substitute
Structural	☐	☐	☐	☐	☐
Strut/Onlay	☐	☐	☐	☐	☐
Morselized	☐	☐	☐	☐	☐

Bone Graft Used—Acetabulum ☐ Yes ➔ ☐ No 🡣

Check ALL that apply:

	Autograft: Iliac crest	Autograft: Femoral head	Autograft: Other	Allograft	Bone Graft Substitute
Structural	☐	☐	☐	☐	☐
Strut/Onlay	☐	☐	☐	☐	☐
Morselized	☐	☐	☐	☐	☐

Legend of Manufacturer Codes:

01 Biomet
02 Ceraver
03 J&J/DePuy
04 Sulzer/Centerpulse
05 Zimmer
06 Wright Medical
07 Smith & Nephew Richards
08 Stryker/Osteonic/Howmedica
99 Other

Formulaire disponible en français
